# Knowledge, attitude and practice of the smear test and its relation with female age**^1^**

**DOI:** 10.1590/1518-8345.0700.2699

**Published:** 2016-06-14

**Authors:** Nara Sibério Pinho Silveira, Camila Teixeira Moreira Vasconcelos, Ana Izabel Oliveira Nicolau, Mônica Oliveira Batista Oriá, Patricia Neyva da Costa Pinheiro, Ana Karina Bezerra Pinheiro

**Affiliations:** 2 Mestrando, Departamento de Enfermagem, Universidade Federal do Ceará, Fortaleza, CE, Brasil; 3 PhD, Professor Adjunto, Departamento de Enfermagem, Universidade Federal do Ceará, Fortaleza, CE, Brasil.; 4 Doutorando, Departamento de Enfermagem, Universidade Federal do Ceará, Fortaleza, CE, Brasil.; 5 PhD, Professor Associado, Departamento de Enfermagem, Universidade Federal do Ceará, Fortaleza, CE, Brasil.

**Keywords:** Health Knowledge, Attitudes, Practice, Age Distribution, Papanicolaou Test, Uterine Cervical Neoplasms, Nursing, Health Education

## Abstract

**Objective:**

to verify the association among the knowledge attitude and practice of women in relation to the smear test and the age range.

**Method:**

a cross-sectional research was undertaken, associated with the knowledge, attitude and practice survey at a Primary Health Care service. The sample consisted of 775 women, distributed in three age ranges: adolescent, young and elderly.

**Results:**

although high rates of inappropriate knowledge were found in all age ranges, it was significantly higher among the adolescents (p=0.000). A similar trend was found in the attitude component, with percentages of inappropriateness in adolescence that drop as age advances (p=0.000). Nevertheless, no statistical difference among the groups was found in terms of practice (p=0.852).

**Conclusion:**

the study demonstrated a relation between the age range and knowledge, attitude and practice of the smear test.

## Introduction

Cervical cancer (CC) is associated with infection by the HPV (Human Papillomavirus), especially subtypes 16 and 18, currently representing an important public health problem. Despite high levels of potential prevention and cure when diagnosed early, this cancer has been appointed as one of the most important concerns globally.

In the Brazilian context, it is considered the third most frequent tumor in the female population and the fourth cause of cancer-related death in women. For 2014, 15,590 new cases were estimated^1^.

In view of the high incidence and mortality related to CC in Brazil, the implementation of effective strategies to control this cancer is justified, including health promotion, prevention, early detection, treatment and palliative care actions^2^.

Health promotion is considered one of the most important pillars to change this epidemiological profile, as its concept considers improvements in quality of life and values the community as a protagonist in this change process. Therefore, Health Promotion is considered a process aimed at expanding the potentials of individuals and communities to act on health determinants that interfere in their quality of life^3^.

Concerning essential services, the supply of the smear test for the early detection of CC is fundamental. The early detection strategies (secondary prevention) are early diagnosis (approach of people with signs and/or symptoms of the disease) and screening (cervical smear test). This test, also known as Papanicolaou, is intended to identify lesions that suggest cancer. Screening for CC is based on the natural course of the disease and on the acknowledgement that invasive cancer evolves from precursor lesions (e lesões precursoras (high grade squamous intraepithelial lesions and *in situ* adenocarcinoma) that can be detected and appropriately treated, impeding the progression to cancer^2^.

Despite the initiatives mentioned, in practice, the application of the smear test has met some barriers, present in a wide range of aspects of women's lives, making it difficult to achieve the desired coverage^4^.

Some studies have discussed the knowledge, attitude and practice of women with regard to the test^5^ ^-^ ^6^, demonstrating high rates of inappropriateness in these three areas. Nevertheless, this assessment was not done in any case by comparing the women in the different age ranges (adolescent, young and elderly).

Thus, the objective in this study was to verify the association among the knowledge attitude and practice of women in relation to the smear test and the age range. Investigating the three components mentioned can indicate the diagnostic conjuncture on possible factors intervening in compliance or not with the test. This assessment can also support the development of future policies and educational strategies to facilitate the preventive approach of CC, adapted to the peculiarities experienced in the different age ranges.

## Method

This cross-sectional research is associated with the KAP (Knowledge, Attitude and Practice) survey for the smear test and was developed between September 2011 and February 2012, involving women attended at a Primary Health Care (PHC) service located in a neighborhood on the outskirts of Fortaleza, CE. At the service, four Family Health Strategy teams (FHS) are active, covering a group of approximately 30 thousand people.

The study population included the women who took the CC prevention test at that service. A convenience sample was selected, with the following inclusion criteria: previous onset of sexual activity and CC prevention test during the data collection period.

Before the test, for all women who agreed to participate in the study, the KAP survey was applied, consisting of pre-coded questions and some open questions to assess the knowledge, attitude and practice in relation to the smear test. This tool was subject to face and content validation^5^.

The knowledge, attitude and practice were assessed according to the criteria described next.

*Appropriate knowledge -* when the woman indicated she had already heard about the test, knew that it served to detect cancer in general, or specifically cervical cancer, and she could mention at least two forms of care needed before undergoing the test.

*Inappropriate knowledge* - when the woman indicated she had never heard of the test or she had already heard of it but indicated not knowing that it served to detect cancer; or when she could not mention at least two types of care needed before undergoing the test.

*Appropriate attitude* - when the woman indicated CC prevention as the motive for undergoing the smear test. When she referred the fact that it is a routine test or the desire to know if everything was alright with her, this was only considered an appropriate attitude when, at the same time, she had appropriate knowledge on the test.

*Inappropriate attitude* - when the woman presented other motivations for undergoing the test than CC prevention.

*Appropriate practice* - when the women had undergone her most recent preventive test three years earlier at most, had returned to get the final result of the test and tried to make an appointment to show the test result.

*Inappropriate practice* - when the women had undergone her most recent preventive test more than three years earlier or had never undergone the test, despite having started sexual activity more than one year earlier, or when she had not returned to get the final result of the test or did not try to make an appointment to show the test result.

In total, 802 KAP surveys were collected. Nevertheless, 27 women had to be excluded from the sample because, for some personal reason, they had not undergone the smear test, totaling a sample of 775 patients.

After the test, all women had a return appointment scheduled with the researcher about 40 to 50 days after the test, at times scheduled for their convenience. Thus, besides the practice before the test, during the KAP survey, compliance with the return could also be observed.

To assess the influence of the age range on the knowledge, attitude and practice regarding the smear test, the women were allocated to three groups: adolescent (up to 19 years), young (20 to 59 years) and elderly (over 60 years). The data were compiled and analyzed using the Statistical Package for the Social Sciences (SPSS), version 20.0.

First, the numerical variables were assessed for normality using the Kolmogorov-Smirnov test. As the distribution of the variables was abnormal, the non-parametric Kruskal-Wallis test was used to compare the numerical variables among the three groups and Pearson's chi-square test to compare the categorical variables. For all analyses, significance was set at 5%.

Compliance with the Brazilian National Health Council standards for research involving human beings was guaranteed. Initially, authorization was requested from the Coordination Office for Permanent Education of the Fortaleza Municipal Government for the development of this study. Next, the study was forwarded to the Research Ethics Committee at Universidade Federal do Ceará, receiving approval under Protocol 81/09.

All participants were informed on the study objectives and, when they agreed, they signed the Free and Informed Consent Form. Anonymity in the information disclosure and freedom to participate in the study or not were guaranteed. For the adolescents to participate in the study, consent was also requested from the legal caregivers (father or mother), present at the service, to involve them in the gynecological appointment.

## Results

The age range of the women investigated in this study varied between 13 and 78 years (mean=35.2 years; Standard Deviation - sd=13.58), education ranged between 0 and 15 years (mean=7.1; sd=3.7) and the onset of sexual activity happened between the ages of 11 and 39 (mean=16.7; sd=3.3). Most of the women who underwent the test during the research were younger than 35 years (58.5%), lived with their partner (69.4%), did not have a paid job (62.3%) and lived near the health service (94.2%).

In the entire sample (775 women), 11.62% were adolescents, 74.45% were young and 13.94% were elderly. The medians and percentages of the age groups are displayed in Table 1.

**Table 1:** Sample distribution according to sociodemographic data. Fortaleza, CE, Brazil, 2012

	Adolescent (n=90/11.6%) Md	Young (n= 577/74.4%) Md	Elderly (n= 108/13.9%) Md	p
Age (years)	17.0	33.0	62.0	-
Education (years)	9.0	9.0	4.0	0.000*
Onset of sexual life (OSL)	15.0	16.0	18.0	0.000*
	%	%	%	
Married/fixed partner	50.0	75.9	50.9	0.000^†^
Lives nearby	93.3	93.9	96.3	0.587^†^
Paid job	27.8	42.5	20.4	0.000^†^
First test	40.0	6.8	0.9	0.000^†^

*Kruskal Wallis; †Pearson's chi-square test;

Regarding education, there is a clear different in years of education among the groups that participated in the research (p=0.000), with a lower level among the elderly (m=4.0). The median age of onset of the sexual activity differed among the groups (p=0.000) and was lower among the adolescents (m=15.0). As regards the marital situation, a significant difference was detected among the medians (p=0.000). The young population showed the highest level of fixed partners (75.9%). Concerning the housing aspect, with regard to the distance from the health service, there was no significant difference (p=0.587), as almost 100% of all women in the study lived near the service. This variable needed to be assessed because, due to the difficulty to access health services, and more specifically the smear test, some people not covered within the area of the PHC service end up having their test there.

Also regarding the sociodemographic data, the situation among the women concerning having a paid job showed a significant converging result (p=0.000), in which the young people reached a higher percentage (42.5%). It is highlighted that 0.9% of women over 60 years of age underwent the test for the first time, in comparison with 40% of women up to 19 years of age.

**Figure 1:**
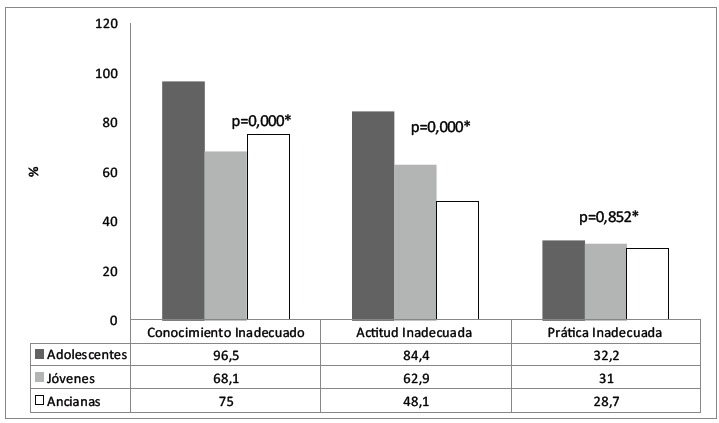
Distribution of knowledge, attitude and practice survey on smear test per age range. Fortaleza, CE, Brazil, 2012

When relating the knowledge, attitude and practice of the smear test with the age range (Figure 1), the group of adolescents stood out with high percentages of inappropriateness.

*Pearson's chi-square test

Although high rates of inappropriate knowledge were found in all age ranges, it was significantly higher among the adolescents (p=0.000), reaching approximately 100% of this group.

Despite lower percentages of inappropriate attitude than inappropriate knowledge, these levels are high during adolescence and drop as age advances (p=0.000).

Lower percentages of inappropriateness were found for practice when compared to the other percentages. Despite a drop as age advanced, according to Figure 1, the different was not statistically significant (p=0.852).

**Figure 2:**
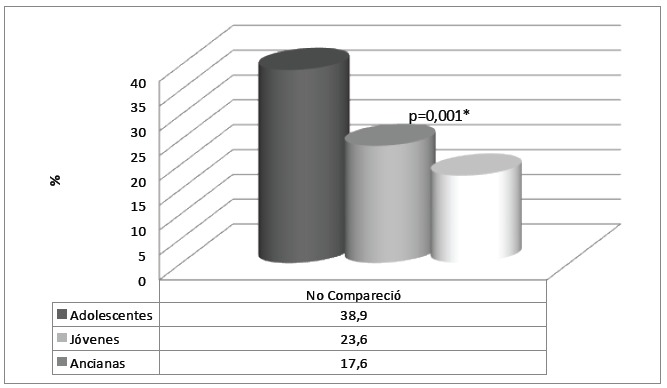
Distribution of women who did not return to receive the smear test result, per age range. Fortaleza, CE, Brazil, 2012

The data related to the women's lack of return to receive the smear test result before participating in the research (Figure 2) revealed that the group of adolescents most frequently did not return (p=0.001). When an appointment was made for this end, during the research, 38.9% of this group did not come to the service.

*Pearson's chi-square test

## Discussion

Many factors influence the conjuncture and epidemiological magnitude of the CC, such as the problems related to knowledge and empowerment of women regarding their attitudes towards the control of this cancer. The motives that make some women not undergo the smear test periodically include lack of education, lack of a partner, younger and older women, lack of time, difficult access to the health service, fear of engagement and constraint. Service-related characteristics, such as distance from the user, lack o material resources for the test, transportation difficulties and bureaucratic aspects also act as barriers for the test^7^ ^-^ ^8^. Another study highlights that 51% of the interviewed women declared that the test should only be done when the woman has gynecological symptoms^9^.

In a study undertaken at a PHC service to assess the knowledge, attitudes and practice concerning the smear test, only 40.4% of these women had appropriate knowledge (knew that it served to detect cancer), only 28% had an appropriate attitude (cited CC prevention as the motive for having the test) and 67.6% were classified as appropriate practice (knew the correct time interval for the test and returned to receive the result)^5^.

These research data reveal that the youngest age range is significantly related with inappropriate knowledge and attitude towards the test. It was evidenced that, in the group of adolescents, inappropriateness concerning the three aspects assessed predominated, calling attention to the development of educative activities focused on this population.

Nevertheless, education levels are higher in the adolescent and young group; by visiting the health service, the adolescents clearly reveal a mistaken motivation to undergo the test (for other reasons), when their main objective should be the early detection of precursor lesions of CC.

A study involving 223 adolescents between 14 and 19 years of age revealed that, among the girls who were already sexually active, 45.8% did not present appropriate knowledge on the prevention test. In addition, 52.5% did not demonstrate information on the HPV infection and its possible consequences, mainly regarding its oncogenic potential. During adolescence, there is a greater possibility of this viral infection turning into a chronic process, which would imply a greater risk of developing cervical cancer^10^.

More than offer the test alone, it should be acknowledge that the women, mainly in the adolescent phase, need further clarifications on the importance of the test to detect CC early, as well as information on the etiology of the disease, centered on the risks of exposure to STD, including HPV. It is fundamental to engage them as protagonists in the education process with a view to promoting a better quality of life.

In that perspective, health promotion goes beyond health care. It is emphasized that people need to get the opportunity to act in the construction process of strategies for this promotion and that they need effective training to control the determinant factors that influence their health^11^.

In addition, it is important for the primary care team to engage in an active search, which is the particular role of community health agents, through home visits or even other communication media, such as telephone use. In addition, educational strategies should be valued that go beyond the restricted spaces of the health service. Actions to promote adolescent health should be considered fundamental in the environments they circulate in.

The school space is a privileged scenario to welcome the adolescents and share decisions and responsibilities with social entities committed to the elaboration of strategies, with a view to reducing the vulnerability^10^.

Another important finding in this research was the Onset of Sexual Life (OSL) around the age of 16 years, confirming the trend that women are exposed to sexually transmitted diseases increasingly early.

HPV is appointed as the main factor in the oncogenesis of CC. Nevertheless, several risk factors can be associated with this tumor, influencing the regression or persistence of this virus, such as: early onset of sexual life, multiple sexual partners, multiple births, use of oral contraception, smoking, immunosuppression, inappropriate intimate hygiene and low socioeconomic condition^2^.

Concerning the marital status (fixed partner), greater stability was observed in the young population (75.5% of the group), showing that the women at the extreme ends of the age range (adolescents and elderly) become more vulnerable to HPV. That is the case because, the earlier the onset of sexual activity (adolescent), the greater the chances of having multiple partners, increasing the vulnerability of this group. On the other hand, the natural course of the disease permits a long interval between HPV infection and manifestations of CC, which explains the high incidence among elderly women.

Having multiple partners exposes women more to HPV infection when compared to women who had a single partner within more than one year, representing a risk factor for HPV^12^.

Another study that was intended to characterize the prevalence and distribution of HPV types among Jamaican women and explore the risk factors associated with HPV infection revealed that the prevalence of this virus was higher in the population between 16 and 19 years of age, among single women and women with more than three sexual partners^13^.

As regards the variable living nearby, no important relation was found in terms of knowledge. Almost 100% of the groups lived near the health services but demonstrated a lack of information. This fact arouses questions as to whether the professionals involved in CC control are engaging in educative actions.

Primary health care is considered the preferred entry door to the health service network. The family health teams are responsible for the care coordination and longitudinal monitoring of the users living in the service's coverage area. Many actions take place at that care level. For the sake of CC control, actions focused on STD prevention are needed, as well as actions focused on the early detection of this cancer, including information and clarifications to the population about screening.

Another important strategy in CC prevention is vaccination against HPV. In view of the profile of the adolescent population, in primary health care, the Ministry of Health incorporated immunization of girls between 11 and 13 years of age in 2014, and between 9 and 11 years of age in 2015. In line with the vaccination campaigns, educative actions need to strengthen other prevention forms, such as consistent condom use and the smear test. After all, the vaccine does not offer total protection against all oncogenic subtypes of HPV. The broader approach, i.e. not restricted to vaccination, converges to the consideration of health promotion in accordance with the integral care principle^14^.

The fact that 40% of the adolescent population investigated was having the test for the first time and had been classified as the group that most demonstrated inappropriate knowledge strengthens the importance of developing specific educative strategies for this population. Another source of concern was that this group was the most absent from the return appointments to get the results.

Simply undergoing the test is insufficient. The women need to understand its importance and return to the service for follow-up. Monitoring, integrality and continuity of care are fundamental to effectively combat the CC^7^.

The care model should be organized to guarantee the access to the services and to integral care, articulating the resources at the different care levels. To influence the determinant factors of CC control actions, it is fundamental for the women's health care to be based on a multiprofessional team and interdisciplinary practice, involving, among other interventions, health promotion^2^. To achieve this promotion, axes should be considered and articulated, such as the construction of healthy public policies, the creation of favorable environments, the strengthening of community action, the development of personal skills and reorientation of health services^11^.

## Conclusion

The study demonstrated a relation between the age range and knowledge, attitude and practice of the smear test. The adolescents were the group with the highest proportions of inappropriate knowledge, attitude and practice. The acknowledgement of the importance of having the test (appropriate attitude) and its appropriate practice improve as age advances.

As a contribution, the study showed the situational diagnosis of the strengths and weaknesses of each age range concerning CC screening, which should be taken into account in the construction of effective strategies, developed by nurses, to increase compliance with the smear test.

Concerning the study limitations, the use of convenience sampling permitted the irregular distribution of the number of women per group, reducing the possibility of inferences on the data found. Research with proportionally comparative groups and longitudinal studies are suggested to assess the impact of primary care professionals' more present and ongoing activities in the school context, with a view to adolescents' adoption of health behaviors, mainly regarding the smear test.
